# Pharmacological and Metabolic Significance of Bile Acids in Retinal Diseases

**DOI:** 10.3390/biom11020292

**Published:** 2021-02-16

**Authors:** Alice Win, Amanda Delgado, Ravirajsinh N. Jadeja, Pamela M. Martin, Manuela Bartoli, Menaka C. Thounaojam

**Affiliations:** 1 Department of Ophthalmology, Medical College of Georgia, Augusta University, Augusta, GA 30912, USA; awin@augusta.edu (A.W.); amdelgado@augusta.edu (A.D.); pmmartin@augusta.edu (P.M.M.); mbartoli@augusta.edu (M.B.); 2 Department of Biochemistry and Molecular Biology, Medical College of Georgia, Augusta University, Augusta, GA 30912, USA; rjadeja@augusta.edu; 3 James and Jean Culver Vision Discovery Institute, Medical College of Georgia, Augusta University, Augusta, GA 30912, USA

**Keywords:** bile acids, UDCA, TUDCA, gut microbiota, ocular diseases

## Abstract

Bile acids (BAs) are amphipathic sterols primarily synthesized from cholesterol in the liver and released in the intestinal lumen upon food intake. BAs play important roles in micellination of dietary lipids, stimulating bile flow, promoting biliary phospholipid secretion, and regulating cholesterol synthesis and elimination. Emerging evidence, however, suggests that, aside from their conventional biological function, BAs are also important signaling molecules and therapeutic tools. In the last decade, the therapeutic applications of BAs in the treatment of ocular diseases have gained great interest. Despite the identification of BA synthesis, metabolism, and recycling in ocular tissues, much remains unknown with regards to their biological significance in the eye. Additionally, as gut microbiota directly affects the quality of circulating BAs, their analysis could derive important information on changes occurring in this microenvironment. This review aims at providing an overview of BA metabolism and biological function with a focus on their potential therapeutic and diagnostic use for retinal diseases.

## 1. Introduction

Bile acids (BAs) are hormones which serve many different roles in the body’s digestive and metabolic systems. Their unique chemical composition contributes to their reported anti-inflammatory and antioxidant effects, explaining their historical use in traditional Chinese medicine (TCM) [1,2,3]. A total of forty-four different animal bile products isolated from both vertebrates and invertebrates have been used to treat ailments of the liver, skin, and biliary system. The earliest records suggest that bile salts from dog and ox were first used as therapeutics; however, the use of bear bile continues to be the most popular and of particular interest in today’s exploration of BA-based therapy [1,2]. Bile and gallbladders collected from black (*Selenarctos thibetamus*) and brown (*Ursus arctos*) bears have been used for thousands of years to remove toxins from the body, stop convulsions, and improve vision [1,3]. In TCM, as a medicine “cool” in nature, bear bile is used to treat “hot” diseases like fever, inflammation, swelling, and pain [1,3]. Modern analysis of bear bile shows that it contains relatively high levels of ursodeoxycholic acid (UDCA) and tauroursodeoxycholic acid (TUDCA) and has numerous pharmacological properties, such as: antimicrobial, anti-inflammatory, anti-convulsant, anti-hepatotoxic, and sedative [3,4,5,6]. The hepatoprotective, anti-gallstones and hypolipidemic effects of bear bile have led to its use as a therapeutic for liver disease and biliary cirrhosis in both TCM and modern Western medicine [2,3,7].

Current interest in BAs arose from the discovery of their signaling properties and ability to regulate the activity of many genes through the orphan nuclear receptor farnesoid X receptor (FXR) [8]. FXR agonists have shown potential as treatments for primary biliary cirrhosis and nonalcoholic steatohepatitis [8,9,10,11]. The Discovery of another BA receptor, transmembrane G-protein-coupled receptor 5 (TGR5), and its widespread distribution in the body, has brought on new explorations on BA-based therapies [8] and their extra-hepatic homeostatic functions on lipid, glucose, and energy metabolism [12,13,14,15]. Interestingly, more recent studies have shown that BAs are protective in neurodegenerative diseases, including a number of ocular afflictions. The aim of this review is to summarize the current knowledge of BA biosynthetic and functional pathways with a particular focus on their effects and therapeutic potential for ocular diseases.

## 2. Bile Acid Physiology and Metabolism

BAs eliminate cholesterol, aid in lipid transport, and stimulate bile flow and biliary phospholipid secretion [16]. BAs maintain liver cells viability while regulating the storage and production of hepatic glucose [17]. Additionally, BAs play a critical role in cholesterol catabolism, being responsible for nearly 50% of its daily turnover [18]. Primarily responsible for lipid digestion and absorption, BAs have been known to act in the small intestine as digestive detergents [18]. Conjugated BAs give rise to bile salts, of which amphiphilic nature allows them to form micelles and work in tandem with lipases at the intestine’s brush border to digest fats [19].

**Figure 1:**
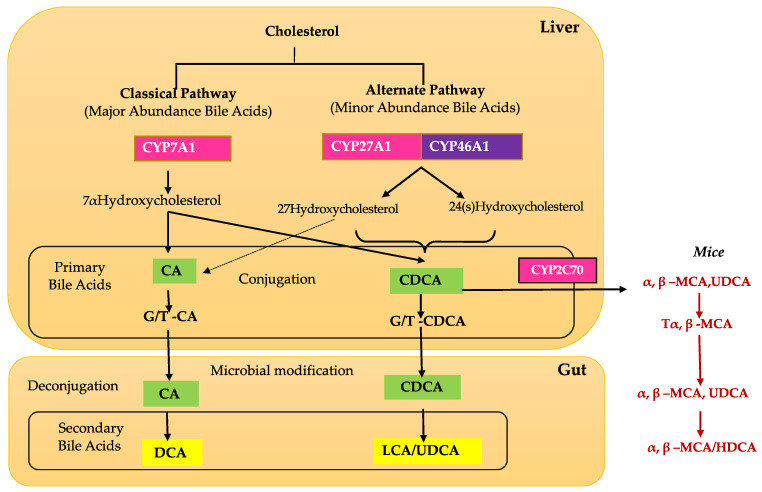
Overview of bile acid metabolism. Bile acids (BAs) are synthesized via two major biosynthetic pathways. In the *classic pathway*, cholesterol is converted to 7α-hydroxycholesterol by the rate-limiting enzyme CYP7A1 (cholesterol 7 alpha-hydroxylase A1). 7α-hydroxycholesterol is ultimately converted into CA (cholic acid) by a sterol 12α-hydroxylase (CYP8B1) or CDCA (chenodeoxycholic acid) without 12α-hydroxylation by CYP8B1. In the *alternative pathway*, cholesterol is first converted to 27-hydroxycholesterol by CYP27A1 (cytochrome P450 Family 27 Subfamily A Member 1), which eventually is converted to CDCA. In the large intestine, bacterial 7α-dehydroxylase removes a hydroxyl group from C-7 and converts CA to DCA (deoxycholic acid) and CDCA to LCA (lithocholic acid). In mouse liver, most of CDCA is converted to α- and β-MCA. In the intestine, bacterial 7α-dehydroxylase activity converts CA and CDCA to DCA and LCA, respectively. CYP3A1 (cytochrome P450 3A1) and epimerase also convert CDCA to the secondary BAs, including THCA (taurohyocholic acid), TMDCA (tauromurideoxycholic acid), ω-MCA (ω-muricholic acid), THDCA (taurohyodeoxycholic acid), and TUDCA (tauroursodeoxycholic acid). LCA and ω-MCA are excreted into feces.

BAs are produced in hepatocytes and are transported to the gallbladder through bile canaliculi. There are two major pathways for BA synthesis: the classical and alternative pathways [16,20] (Figure 1). The classical pathway is initiated by cholesterol 7α-hydroxylase (CYP7A1) and regulated by FXR [14,21,22]. CYP7A1 is the only rate-limited enzyme in BA synthesis and leads to the production of the two primary BAs: cholic acid (CA) and chenodeoxycholic acid (CDCA). In the classical pathway, CYP7A1 is converted to 7 α- hydroxy-4-cholesten-3-one (C4), which is the precursor to both CA and CDCA [14,16,22]. C4 can then be converted to CA with 12α-hydroxylase (CYP8B1), and without this conversion, C4 is converted to CDCA [14,16,22]. Mitochondrial sterol 27-hydroxylase (CYP27A1) catalyzes the steroid side-chain oxidation and subsequent proximal β-oxidation of C4 to form 24-carbon compounds such as CA and CDCA [14,16,22].

The alternate pathway is initiated by sterol 27-hydroxylase (CYP27A1), a mitochondrial cytochrome P450 enzyme that is widely distributed in macrophages and most tissues [14,23,24]. Studies have shown that the alternative pathway contributes to around 9% of total BA synthesis in human hepatocytes [16]. Initiation by CYP27A1, followed by CYP7B1 (25-hydroxycholesterol 7-alpha-hydroxylase), leads to the synthesis of CA and CDCA in the liver and extrahepatic tissues such as steroidogenic tissues and macrophages [16,24].

**Figure 2:**
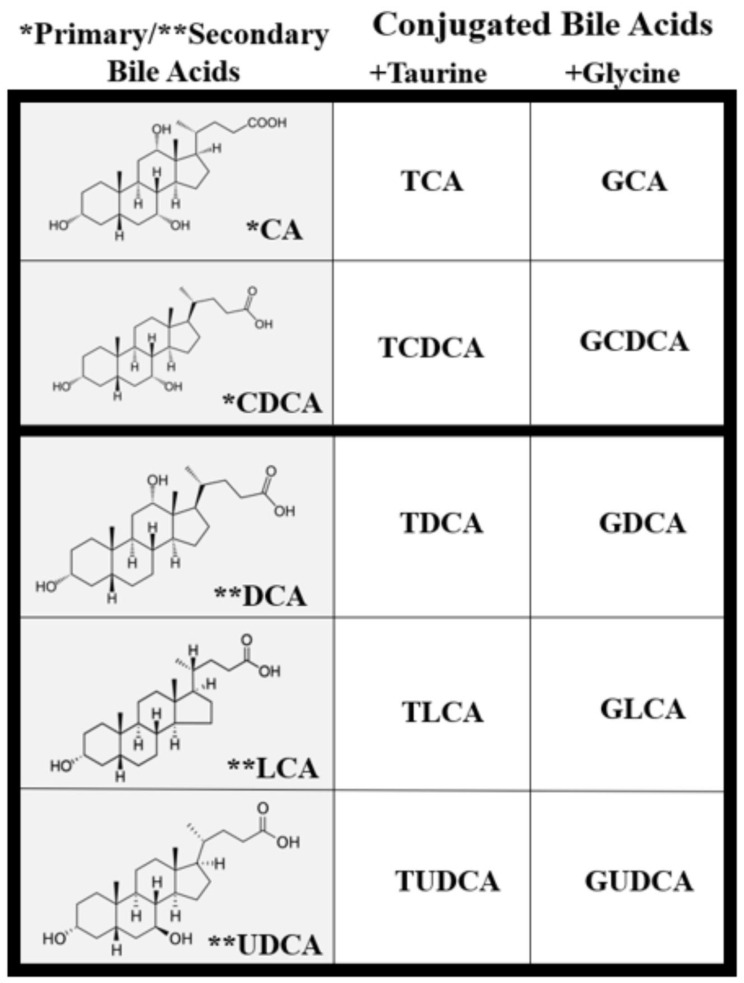
Types of unconjugated and conjugated bile acids. Primary and secondary BAs are either taurine or glycine conjugated to form primary and secondary conjugated BAs.

Once in the intestinal lumen, gut microbiota produces secondary bile salts by modifying primary BAs with 7α-hydroxylation, deconjugation, oxidation, and epimerization of hydroxyl groups on carbon-3, carbon-7, and carbon-12 [21,25,26]. Bile salts, conjugated BAs, are deconjugated via hydrolysis of the amide bond between glycine or taurine and the BA, allowing the BAs to be used as substrates for modification by the microbiota. This reaction is catalyzed by bile salt hydrolases (BSH) in bacterial enzymes [27,28,29] (Figure 2). Dehydratases from the anaerobic gut flora turn unconjugated CA and CDCA into deoxycholic acid (DCA) and lithocholic acid (LCA), respectively, and epimerization of hydroxyl groups of CDCA by hydroxysteroid dehydrogenases of intestinal bacteria leads to the formation of UDCA [30].

Unconjugated BAs are reabsorbed into intestinal enterocytes via passive diffusion in the jejunum and colon, whereas conjugated BAs, bile salts, are reabsorbed through a sodium-dependent BA transporter (ASBT) in the jejunum [27,31]. While 95% of BAs are reabsorbed and return to the liver via the portal circulation, the remaining 5% do not get reabsorbed at the intestinal lumen and are excreted in feces [14,32]. This mechanism puts a constant demand on the liver to continue BA synthesis. Though BAs are recycled through enterohepatic circulation, less than 10% of BAs reach systemic circulation [14], where they can be detected in both plasma and cerebrospinal fluid on the scale of ng/mL [33]. However, BA concentration has never been measured in ocular fluids [21].

## 3. Cytotoxic and Cytoprotective Effects of Bile Acids

Though BAs are an integral part of numerous biological processes, they can also exert toxic effects and cause inflammation, apoptosis, and cell death [16,34,35]. BA concentration and hydrophobicity are important in determining their toxic or protective effects, with a more hydrophobic BA being more toxic. Increased retention of hydrophobic BAs promotes hepatocyte apoptosis or necrosis that may lead to liver injury [34,36,37]. BA hydrophobicity is determined by the number, position, and orientation of its hydroxyl groups along with amination at its C-24 position and, based on this, the BA sequence from least to most hydrophobic is: UDCA, CA, CDCA, DCA, and LCA [34,38] (Figure 2). Once a BA is conjugated, however, it becomes more hydrophilic and, therefore, less cytotoxic [34,38]. Defective transport, faulty biosynthesis, or excess bacterial degradation may lead to an excess of toxic BAs. Specific conditions associated with high intracellular and extracellular concentrations of BAs include cholestasis and BA malabsorption, respectively [27]. Disorders associated with dysregulated BA metabolism include cholestatic liver disease, dyslipidemia, fatty liver diseases, cardiovascular diseases, and diabetes, among others [16,39,40].

Studies have shown that the accumulation of hydrophobic BAs inside hepatocytes leads to their injury and death. In one study, hepatocytes expressed dramatic levels of subcellular damage when exposed to concentrated CA and CDCA [34]. The canalicular membranes were particularly altered in the presence of concentrated LCA and tauro-LCA [36], the most hydrophobic of the common BAs. Moreover, biliary epithelium cells were very sensitive to the cytotoxicity of hydrophobic unconjugated bile salts while they remained largely undamaged by taurine- or glycine-conjugated bile salts [36].

Aside from their hydrophobicity, abnormal amounts of BAs can lead to toxic effects on hepatic cells as well. Decreased BA concentration will stimulate hepatic cell apoptosis, while increased BA concentration will stimulate necrosis [34,41]. Cytotoxic BAs were shown to alter membrane cholesterol levels, whereas cytoprotective BAs had no effect on the membrane. This evidence suggests that BA cytotoxicity could depend on their effects on plasma membrane integrity or on mitochondrial membrane affinity for apoptotic proteins [42]. BAs have also been shown to induce injury on several cell types, including hepatic, biliary, gastrointestinal, renal, and lung cells [34]. Interestingly, patients experiencing the cytotoxic effects of hydrophobic BAs might undergo hydrophilic BA-based therapy, which entails displacing cytotoxic BAs with the non-toxic UDCA or TUDCA.

UDCA and TUDCA, although present at very low circulating levels in humans, have been studied for their cytoprotective effects due to their hydrophilic nature and low toxicity [43]. Their mechanism of action is not fully understood; however, stabilization of cell membranes (structure and fluidity), protection against oxidative injury, and prevention of apoptosis have all been reported [34,43].

BA pharmacological use has been applied to pathologies such as cholelithiasis, primary biliary cirrhosis, primary sclerosing cholangitis, and cystic fibrosis and is being suggested for neurodegenerative disorders associated with increased apoptosis such as Huntington’s Disease [34,44]. The most significant effects of UDCA and TUDCA as therapeutic agents have specifically been studied in patients with either liver or digestive disease [2]. For example, UDCA is used in patients with cholestatic liver disease to slow disease progression by decreasing hepatocyte injury. Moreover, it can be used in patients with BA malabsorption to sequester BAs and provide symptomatic relief from diarrhea [45].

Some studies demonstrate that the mechanism by which UDCA protects membranes is dependent on membrane cholesterol. In one study, cholesterol was found to influence the toxicity of both UDCA and the highly toxic sodium deoxycholate (SDC). UDCA was able to mitigate the cytotoxic effects of SDC only in the presence of cholesterol, whereas, enhancing its cytotoxic effects in the absence of cholesterol. This relationship of events suggests that the extent of UDCA cytoprotective effects is dependent on the level of cholesterol in membranes. In the context of treating cholestasis patients with UDCA, one must consider changes in membrane cholesterol composition that might affect the efficacy of this drug [37].

## 4. Retinal Bile Acid Metabolism

Although the liver is the primary site of BA production, several recent studies have identified extrahepatic production sites, including the brain and retina [14,21]. The BA pathway plays an important role in cholesterol metabolism in the retina. Emerging evidence and our unpublished data show that unlike the liver, the brain and retina mainly rely on the alternate BA biosynthetic pathway [14]. The first step in the alternate pathway is initiated by the conversion of cholesterol into 27-hydroxycholesterol via CYP27A1. Subsequently, oxysterol 7α-hydroxylase (CYP7B1), through subsequent hydroxylation, leads to the formation of primary BAs, CA, and CDCA (Figure 1). Simultaneously, the formation of 24(S)-hydroxycholesterol from cholesterol is carried out by CYP46A1 (cholesterol 24-hydroxylase). 24(S)-hydroxycholesterol is then metabolized via oxysterol 7α-hydroxylase II (CYP39A1) to generate CDCA (Figure 1). Cholesterol involvement is implicated in several retinal pathologies, such as in age-related macular degeneration, where large cholesterol-rich deposits are a hallmark feature [46]. CYP27A1 and CYP46A1 are two enzymes that are important to ocular function in humans. In the retina, CYP27A1 is the principal hydroxylase that catalyzes the hydroxylation of cholesterol for BA synthesis to account for the majority of enzymatic cholesterol elimination in the retina [46]. Deficiency in CYP27A1 is seen in *cerebrotendinous xanthomatosis*, a rare congenital disease characterized by several neurological and ocular symptoms such as cataracts, neurological dysfunction, and premature retinal degeneration [46]. One study found an increase in focal cholesterol deposits along Bruch’s membrane in CYP27A^-/-^ mice [46]. Combined deficiency in CYP27A1 and CYP46A1 results in neovascularization and lipid accumulation in the mouse retina [47]. CYP46A1 is expressed in both the brain and the retina, though mainly the neural retina, and has a role in maintaining retinal cholesterol homeostasis and retinal immune response [48]. A single nucleotide polymorphism in the CYP46A1 gene, rs754203, is a risk factor for glaucoma and has also been identified as a predictive marker for the risk of cataracts [49]. Based on the current literature, it is evident that the retinal BA pathway is understudied, and more studies are required to fully appreciate and understand BA retinal metabolism and functions.

## 5. Bile Acid Transporter and Receptor in the Retina

BAs require essential transporters to maintain their enterohepatic circulation and facilitate their active transport through membrane barriers in the liver and intestine. The sodium BA cotransporter family (SLC10) is a solute carrier family of genes that has been found to contain several BA transporters localized in many different tissues in the body. Most studied are the Na^+^/taurocholate co-transporting polypeptide (NTCP; SLC10A1) and the apical sodium-dependent BA transporter (ASBT; SCL10A2), which serve their primary transport functions in the liver and intestine, respectively. The gene SLC10A4 has been localized in the human eye, and it is associated with cholinergic synapses throughout the central nervous system. While SLC10A4 is also responsible for encoding protein P4, little data supporting the functional properties of the P4 gene is currently available [50,51].

Emerging evidence suggests that aside from their conventional biological function in the enterohepatic system, BAs are also important signaling molecules [16] by activating specific receptors, such as FXR and TGR5, and their downstream signaling cascades (Table 1). In the retina, the FXR and TGR5 receptors are currently the most studied, however, reports on FXR retinal expression remain inconsistent, and TGR5 expression has been reported only in the adult retina [21,52,53,54]. Other BA receptors have been identified, such as vitamin D receptor (VDR), pregnane X receptor (PXR), glucocorticoid receptor (GR) and mineralocorticoid receptor (MR), constitutive androstane receptor (CAR), α5 β1 integrin, and sphingosine-1-phosphate receptor 2 (s1PR2) [55,56]. The biological functions of these receptors are much less characterized and have not been previously studied in the retina. Furthermore, the literature describing the importance of BA receptors in a wide range of diseases of the liver, biliary system, and gastrointestinal tract is vast [57], whether their extrahepatic functions are underinvestigated. No current information is available on BA receptors expression pattern in the developing retina.

## 6. Pharmacological Effects of Bile Acids in Ocular Disease

In the last decade, studies on the usefulness of BAs in the treatment of ocular diseases have gained great interest in the scientific community. Particularly, UDCA and TUDCA are reported to be beneficial against photoreceptor degeneration, ganglion cell death, cataract, diabetic retinopathy (DR), etc. [21]. Previously, the neuroprotective properties of BAs in retinal diseases have been reviewed by multiple authors [58,59,60]. Herein we summarize recent advances in using BAs to treat ocular diseases and highlight significant lacunae and future studies (Table 2). Additionally, our current review also discusses the recent studies, including ours, on the effects of BAs and their receptor agonists in the retinal vasculature in various forms of retinal diseases, including DR and retinopathy of prematurity (ROP). Of interest, our review also focuses on the role of the BA-gut microbiome axis in various retinal diseases, a topic that was not discussed in previously published review articles.

Although the mechanism of action of UDCA and TUDCA remains unclear, it is thought to involve suppression of both caspase-dependent and caspase-independent apoptosis, likely by inhibiting the release of the apoptosis-inducing factor (AIF) from the retinal pigment epithelium (RPE), photoreceptors, and retinal ganglion cells (RGC) [21,61,62].

Retinal detachment, oxidative stress, and inflammation are also a key contributor to photoreceptor degeneration following their detachment from the RPE [63]. Photoreceptor cell death and retinal detachment are associated with numerous ocular diseases such as ROP, retinitis pigmentosa, age-related macular degeneration, and DR [63,64,65,66,67]. A study by Mantopoulos et al. showed that TUDCA preserved photoreceptor survival after retinal detachment by inhibiting caspase activity and reducing ER and oxidative stress [68].

In galactosemic rats, TUDCA treatment alleviated cataract formation by inhibiting the unfolded protein response (UPR)-dependent pathway [69]. Cataract formation results from stressors leading to ER and oxidative stresses of lens epithelial cells (LECs), resulting in a UPR-dependent death pathway. TUDCA rescued cultured human LECs that were treated with ER stressors. The same group also reported that galactosemic rats treated with TUDCA had significantly reduced LEC death and partially delayed hyper mature cataract formation [69]. However, in selenite-induced nuclear cataract in rats, a control demonstrating the UPR-independent death pathway, TUDCA did not prevent LEC death and did not alleviate cataract formation. Overall, TUDCA showed potential as a prophylactic drug for some types of cataracts in which ER stress and LEC death play a central role [69]. When the ER is subjected to multiple pressures, unfolded proteins accumulate within the organelle, stimulating the UPR. The consequences of this response are ultimately excessive ER stress and cell death [70,71].

One study has explored the significance of TUDCA structural resemblance to compounds that bind to a light-activated form of rhodopsin to inhibit the activation of transducin. This study used computer modeling systems to show that TUDCA docks to light-activated rhodopsin using a similar mode of binding to the C-terminal domain of the transducin alpha subunit. These results suggest that TUDCA specific interaction with rhodopsin may contribute to its effect as a potential therapeutic compound for degenerative retinal diseases [53].

Retinal degenerative diseases are important causes of blindness with little or no present effective cures. BA therapeutic potential has been tested in several models of retinal degeneration. Leber congenital amaurosis (LCA) is a form of retinal dystrophy that often leads to severe vision impairment and blindness; it has been shown that ER stress induced by S-opsin aggregation leads to rapid cone degeneration [72,73,74]. TUDCA has been shown to reduce ER stress and apoptosis in mice models of LCA and significantly preserve photoreceptors survival [62,73].

In one model of light-induced retinal damage, TUDCA treatment was shown to reduce superoxide radical levels [75]. TUDCA treated mice were found to have significantly higher a- and b-wave amplitudes on ERG readings and presented with milder retinal functional damage seven days post-light exposure when compared to mice given other treatments [75]. Similar findings were also shown in a model of light-induced retinal degeneration (LIRD). Mice treated with TUDCA showed greater ERG a-wave and b-wave amplitudes compared to control mice. Moreover, TUDCA-treated mice were found to have thicker outer nuclear layers, more photoreceptor cells, and more fully developed photoreceptor outer segments [76]. Collectively, these results indicate that TUDCA slows retinal degeneration.

In models of retinitis pigmentosa (RP), TUDCA has also been studied as a potential therapeutic agent. The common outcome of the RP disease family includes apoptosis of rods followed by degeneration of cones [77,78,79]. One of the main causes of RP is a mutation in the GTPase regulator (RPGR) gene, which codes for a scaffold protein responsible for ciliary protein trafficking processes [80]. In one study, TUDCA was found to suppress microglial activation preceding retinal degeneration in an RPGR conditional knockout mouse model [80]. Additionally, TUDCA treatment inhibited inflammation and prevented photoreceptor degeneration. Together, these findings support TUDCA as a potential therapeutic tool for RPGR-associated RP.

Boatright et al. utilized *rd10* mice to test the efficacy of TUDCA in RP [76]. Rd10 mice have a missense mutation in phosphodiesterase 6B, leading to rod degeneration around postnatal days 14-16 (P14-P16). Their results showed that systemic injection of TUDCA prevented apoptosis during the early stage of the disease and preserved function and morphology of photoreceptor cells in these mice by inhibiting caspase-mediated photoreceptor apoptosis. [76]. In another study by the same group, TUDCA ability to preserve photoreceptors during retinal degeneration in rd10 mice was further analyzed and confirmed at later stages (P30) when photoreceptor cell loss peaks [81]. At P30 in the rd10 model, the majority of rods have degenerated, and only some cones remain [82] resembling the end-stage RP for most patients [83]. TUDCA treatment between P6 and P30 preserved overall photoreceptors number and function [81] and significantly reduced caspase 3 activation and apoptosis. Systemic TUDCA treatment in these mice was able to delay retinal damage to at least P30. In this case, TUDCA treatments were also able to slow the loss of both photoreceptors and retinal function. Additionally, TUDCA treatment was found to slow the loss of RGC and prevent epithelial cell death [84].

In another study of retinal degeneration, photoreceptor degeneration in mice retinas was chemically (N-methyl-N-nitrosourea) induced [85]. To explore TUDCA as a potential therapeutic agent, TUDCA was subcutaneously injected. Retinal flat-mounts of TUDCA treated mice showed efficient cone photoreceptor preservation. Additionally, TUDCA therapy rectified abnormalities in the visual signal shown to inhibit photoreceptor degeneration and decrease visual impairments in mice that received transmission. A multi-electrode array was used to detect the restrictive effect of TUDCA therapy on spontaneous firing response as well as the supportive role TUDCA therapy plays in enhancing the light-induced firing response. In degenerative retinas, TUDCA treatment was noted to preserve the basic configurations of the ON–OFF signal pathway. The results of this study suggest subcutaneous delivery of TUDCA has therapeutic potential in retinopathies with progressive photoreceptor degeneration [85].

BAs were also tested in models of optic nerve injury. Optic nerve transection was performed on C57BL/6 mice following systemic TUDCA treatment for 10 days. Immunohistochemistry staining on retinal flat mounts indicated a loss of 60% of RGC in control mice, which was completely prevented in mice treated with TUDCA [84].

Diabetic retinopathy, a retinal neurovascular disorder, is the leading cause of blindness in adults of working age groups in the Western world [86,87]. UDCA has also been found to reverse the breakdown of the blood-retinal barrier (BRB) and reduce retinal inflammation in models of DR [88]. Reduced retinal expression of interleukin-1β and interleukin-6 in response to UDCA treatment suggests that UDCA inhibits retinal inflammation and attenuates BRB breakdown. Moreover, UDCA treatment increased expression of the tight junction proteins claudin-1 and claudin-19 and led to a reversal of the reduced thickness of both the inner and outer nuclear layer of the retina [88]. UDCA’s suggested ability to reverse damage to retinal layers typically compromised in ocular disease continues to drive research toward its use as a therapeutic agent for DR.

Pericytes play an important role in maintaining vascular integrity, and it is thought that retinal pericytes loss, an early hallmark of DR, leads to the BRB breakdown [89,90,91,92]. Endoplasmic reticulum stress may be involved in this process [93]. UDCA has also been reported to reduce pericyte depletion in models of DR [93]. UDCA clearly attenuates the increase in ER stress and prevents loss of pericytes and vascular integrity in DR [93]. Another recent study investigated the effects of UDCA in pericyte depletion mice by injection of an antibody against platelet-derived growth factor reception-β (PDGFR-β clone APB5) [94]. UDCA reduced the expression of F4/80+ macrophages in the APB5-induced retina according to immunofluorescent labeling. UDCA also reduced the increased expression of angiogenic factors and inflammatory mediators (vascular endothelial growth factor; VEGF, intercellular adhesion molecule-1, and monocyte chemotactic protein-1). Along with microvascular changes, neural components of the retina are also affected in DR [95,96]. A study by Gaspar et al., 2013, evaluated the neuroprotective effects of TUDCA in high glucose-treated rat retinal neurons. TUDCA was able to prevent cell death induced by elevated glucose through its anti-apoptotic and antioxidant properties in cultured retinal neural cells. Together, these results suggest that UDCA and TUDCA attenuate retinal vascular and neural abnormalities associated with DR [93,94].

TGR5 receptor plays an important role in regulating metabolic homeostasis [15]. TGR5 activation has also been linked to improving retinal function, and TGR5 agonists have been reported to improve biliary epithelial barrier function [97]. One study demonstrated that treatment of Streptozotocin (STZ)-induced diabetic rats with TGR5 agonist INT-777 was protective against hyperglycemia-induced vascular permeability [97]. Another study by Beli et al., 2018 showed that TGR5 activation using INT-767 significantly reduced acellular capillaries and inflammation in the db/db mice. Particularly, INT-767 treatment to diabetic mice significantly reduced the number of acellular capillaries and numbers of macrophages, leukocytes, and activated microglia in the db/db mice retina [52]. Together, these results emphasize that TGR5 activation could be a viable therapeutic strategy to ameliorate DR.

Age-related macular degeneration is known to result in severe visual loss due to the development of choroidal neovascularization (CNV) along with related manifestations, such as subretinal hemorrhage, RPE detachment, and fibrovascular disciform scarring, also known as wet or exudative AMD [98,99,100]. Woo, et al. demonstrated that UDCA and its derivative, TUDCA, when intraperitoneally injected to the laser-treated rat model, could effectively suppress early VEGF elevation in the retina which inhibits the upregulation of VEGF and eventually results in the reduction in CNV size and vascularity, suggesting the possibility of treating CNV [101]. In a recent study, a mouse model of laser-induced CNV was used to test the efficacy of YSB201 (an aqueous solution of UDCA) in the treatment of AMD [102]. Results indicated that the YSB201 treatment inhibited the return of angiogenesis to the normal choroidal tissue condition. Additionally, while YSB201 treatment at 125 mg/kg/day led to the recovery of retinal function post-injury, the higher dose of YSB201 treatment at 250 mg/kg/day did not yield significantly higher recovery effects. The results of the study specifically describe the inhibitory effects of UDCA formulation on CNV with functional recovery in mice retinas [102]. Taken together, these results suggest that UDCA could be used as a potent supplement for the cure of AMD and related retinal complications.

Further evidence of UDCA therapeutic potential was seen in our recent study of ROP, a leading cause of preventable blindness in children [103,104]. We investigated the efficacy of the secondary bile acid, UDCA and its taurine and glycine conjugated derivatives TUDCA and glycoursodeoxycholic acid (GUDCA) in preventing retinal neovascularization (RNV) in an experimental model of ROP. While all three secondary BAs have pharmacological effects, this ROP model found that UDCA was most effective in demonstrating anti-angiogenic effects, preventing loss of neuronal cells, and protecting against reactive gliosis and BRB breakdown while maintaining normal revascularization of the central retina [105]. UDCA was also able to decrease VEGF and inflammatory cytokine expression [105]. Collectively, our results suggest that UDCA could represent a new effective therapy for ROP.

**Table 2:** Pharmacological effects of bile acid treatments on various ocular diseases.

Ocular Disease	Bile Acid /Agonist	Dose	Findings	References
**Leber congenital amaurosis**	TUDCA	Systemic injection; 500 mg/kg b.w./3 days	TUDCA is a potential agent in reducing ER stress, to prevent apoptosis, and preserve cones in the LCA model	[61]
**Retinal detachment**	TUDCA	Intraperitoneal injection; 500 mg/kg b.w.	TUDCA preserves photoreceptors after retinal detachment, inhibits caspase activity, and reduces ER and oxidative stress	[67]
**Cataracts**	TUDCA	Subcutaneous injection; 500 mg/kg body weight (b.w.)/day	TUDCA treatment alleviates cataract formation via the UPR-dependent pathway	[68]
**Oxidative stress-induced retinal degeneration**	TUDCA	Intraperitoneal injection; 500 mg/kg every 3 days	TUDCA produces modest preservation of outer nuclear layer thickness and rod function at P30. And significant preservation of cone cell number and cone function at P50	[74]
**Retinal degeneration**	TUDCA	Subcutaneous injection; 500 mg/kg b.w.	TUDCA greatly slowed retinal degeneration in LIRD, and rd10 mice protected photoreceptor and suppressed apoptosis	[75]
**Rpgr-Associated Retinitis pigmentosa**	TUDCA	Intraperitoneal injection; 500 mg/kg b.w.	TUDCA suppresses microglial activation, inhibits inflammation, and prevents photoreceptor degeneration	[79]
**Retinal degeneration**	TUDCA	Systemically injected; 500 mg/kg every 3 days from P6 to P30	TUDCA-treated rd10 retinas had fivefold more photoreceptors than vehicle-treated retinas. TUDCA treatments did not alter the retinal function or morphology of wild-type mice when administered to age-matched mice.	[80]
**Retinal degeneration**	TUDCA	Subcutaneous injection; 500 mg/kg b.w./ day	TUDCA treatment: reduces caspase 3 activation and apoptosis, slows the loss of photoreceptors and retinal function, and delays retinal damage	[83]
**Retinal degeneration**	TUDCA	Subcutaneous injection; 500 mg/kg b.w.	TUDCA inhibits photoreceptor degeneration and decreases visual impairments. TUDCA rectifies abnormalities in visual signal transmission	[84]
**Diabetic Retinopathy**	UDCA	Oral delivery, intragastric administration; 30 mg/kg b.w.	UDCA reverses the breakdown of the blood-retinal barrier and reduces retinal inflammation	[87]
**Diabetic Retinopathy**	UDCA	Intraperitoneal injection; 100 mg/kg/d b.w.	vascular integrity was improved and pericyte loss reduced in the retina of STZ-induced diabetic mice	[92]
**Diabetic Retinopathy**	UDCA	Subcutaneous injection / daily for P7-9 neonates; 100 mg/kg.	UDCA reduced the increased expression of angiogenic factors and inflammatory mediators (vascular endothelial growth factor, intercellular adhesion molecule-1, and monocyte chemotactic protein-1	[93]
**Diabetic Retinopathy**	INT-777 (semisynthetic bile acid) a TGR5 agonist	50 ng/μL, 5 μL was injected into the vitreous cavity	Upregulation or activation of TGR5 may inhibit RhoA/ROCK-dependent actin remodeling and represent an important therapeutic intervention for DR.	[96]
**Choroidal** **neovascularization (CNV)**	UDCA TUDCA	Intraperitoneal injection; UDCA 500 mg/kg, TUDCA 100 mg/kg,	The systemic administration of UDCA and TUDCA suppressed laser-induced CNV formation	[100]
**Choroidal** **neovascularization (CNV)**	UDCA	Oral delivery; 125 or 250 mg/kg b.w./day	UDCA inhibits CNV and promotes functional recovery in mice retinas	[101]
**Retinopathy of prematurity**	UDCA TUDCA GUDCA	Intraperitoneal injection; 50 mg/kg	UDCA decreased the extension of neovascular and avascular areas, whereas treatments with TUDCA and GUDCA showed no changes. UDCA also prevented reactive gliosis, preserved ganglion cell survival, and ameliorated OIR-induced blood-retinal barrier dysfunction.	[104]

Together, these findings support the idea that BAs have the potential to show their effects at nearly every structural layer of the eye. Evidence continues to support their therapeutic potential in neurodegenerative and ocular disorders.

## 7. Influence of Changes in Gut Microbiota on BA Circulation and Retinal Diseases

The human gut is a densely populated ecosystem containing numerous bacteria that coordinate with the host and facilitate the digestion of exogenous foods and the generation of secondary metabolites. While the bacteria found here are primarily obligate anaerobes, facultative anaerobes, archaea, and yeast also reside in the gut [106]. Bacteria in the largely anaerobic environment of the gut microbiome use sloughed off intestinal cells, plant polysaccharides, starch, cellulose, and bile components as substrates for fermentative metabolism [107]. The composition of the microbiome is fluid and can be affected by a variety of factors, including diet, age, consumption of antibiotics, and disease. Additionally, BA pool and composition have both direct and indirect antimicrobial effects on the gut microbiome [106,107]. Though most BAs are reabsorbed in the small intestine and enter the enterohepatic circulation to be recycled and reused, around 5% of BAs pass through to the large intestine, where they are eventually excreted in the feces [108]. It is in the large intestine where their interactions with the gut microbiome can compromise bacteria and affect the health and physiology of the host [108]. One study recognized that germ-free animal models lack secondary BA production [109]. Another postulates that since BAs are ligands of metabolism-linked bile-responsive receptors, changes in BA composition facilitated by the host microbiota activity may affect their interaction with specific receptors [110].

Once in the large intestine, many intestinal bacteria, specifically those of *Clostridium*, *Bifidobacterium*, *Lactobacillus*, *Bacteroides*, and *Enterococcus* genera, have the ability to deconjugate BAs into primary BAs with BSH, which hydrolyze the BA amide bond [111]. Specifically, species of gut microbes belonging to the genera *Bacteroides*, *Clostridium*, *Lactobacillus*, and *Bifidobacterium* contain genes which encode BSH. It is suggested that BSH activity is used by bacteria as a BA detoxification mechanism, which can alter both the local gastrointestinal and the systemic functions of the host organism [109]. Aside from deconjugation, BAs are also modified through oxidation and epimerization of 3-,7-, and 12-hydroxyl groups, along with esterification and desulfation. These modifications are complete through bacteria of the genera *Bacteroides*, *Eubacterium*, *Clostridium*, *Bifidobacterium*, *Lactobacillus*, *Peptostreptococcus*, and *Escherichia* as well as bacteria of the genera *Clostridium*, *Peptococcus*, and *Fusobacterium*, respectively [111]. Subsequently, selected bacteria of the *Eubacterium* and *Clostridium* genera can transform primary BAs into secondary BAs with 7-α-dehydroxylation [112].

BAs are known to affect many systems in the body, and most studies demonstrate many of these effects can be toxic [58]. The amphipathic nature of BAs and their hydrophobicity have been linked to negative effects, including cell damage, increasing bacterial membrane permeability, and subsequent cell death [108,112]. Though Gram-negative bacteria have been shown to be more resistant to the effects of BAs than Gram-positive, both are vulnerable to the increase in oxidative and pH stress caused by BAs [112]. BAs can also affect gut microbiota indirectly. These effects are mediated by the activation of FXRα [108]. FXR in the intestines regulates several genes, including those for mucosal defense, ileal BA binding protein, and fibroblast growth factor 15 (FGF-15). In one study using mice lacking the gene for CYP27A1, which, as mentioned earlier, is an essential enzyme for BA synthesis, authors found that without an agonist for the gut FXR receptor, there was an overgrowth of both aerobic and anaerobic bacteria in the ileum and colon [113]. Subsequent administration of FXR agonist to the same mice revealed a reversal of the overgrowth by preventing translocation of bacteria across the intestinal mucosal barrier [108,113].

Disruption of the gut microbiome alters BA metabolism and leads to deficits in the metabolic pathways they regulate, including glucose and cholesterol homeostasis; by doing so, they threaten the host’s overall health [112]. Gut microbiome dysregulation and changes in BA profile have been evidenced in patients with Type 2 Diabetes, inflammatory bowel disease, or with other disorders involving low-grade inflammation [112,114,115,116,117]. Changes in the conjunctival microbiome have been observed in patients with Type 2 Diabetes, mirroring the changes associated with the altered gut microbiome. Changes in both the conjunctival and gut microbiomes can influence the progression of DR [118]. One study of the microbiome in db/db mice demonstrated the impact of gut microbiota changes on BA circulation and retinal disease [52]. The study relied on the use of long-term intermittent fasting (IF) to induce changes in the gut microbiome of db/db mice. Compared to controls, mice subjected to IF experienced changes in the gut microbiome that reduced acellular capillaries and leukocyte infiltration. Microbiome analysis of IF mice reflected a drastic change in the Firmicute/Bacteroidetes ratio, indicating that long-term IF had altered the model’s microbiome. It is thought that IF might have led to lower numbers of bacteria that express BSH activity and, thus, an increase in the ratio of conjugated to unconjugated BAs in the model. As expected, IF mice showed significantly increased levels of the neuroprotective TUDCA. While expression of the TGR5 receptor did not change with IF or diabetes, IF reduced TNF-α (Tumor necrosis factor alpha) mRNA, a downstream target of TGR5 activation. Together, the findings of this study suggest that IF restructures the microbiota to favor the production of TUDCA and protect the retina via TGR5 activation [52].

## 8. Conclusions

BAs possess unique properties that make them candidates for the treatment of various diseases characterized by inflammation and oxidative stress. Secondary BAs like UDCA and TUDCA are more hydrophilic and less cytotoxic than their primary BA counterparts, making them a candidate for therapeutic treatment. While UDCA and TUDCA are found at low levels in systemic in humans, they have been applied to pathologies like cholestasis, cystic fibrosis, and primary biliary cirrhosis for their pharmacological qualities. The gut microbiota converts primary BAs to secondary BAs, and current evidence indicates the gut microbiome plays a significant role in BA circulation. Although the retinal BA pathway and BA interactions in the eye are not fully understood, it is known that the gut microbiome and its resulting effects on retinal BAs may play a central role in ocular disease. Future investigation is needed to determine the mechanism by which changes in the gut microbiome affect retinal diseases and how BA therapy can modify this interaction.

## Figures and Tables

**Table 1:** Reported bile acid (BA) receptors expression in various tissues.

Nuclear Receptors	Affinity	Location
FXR	CDCA, CA, LCA, DCA	Liver, intestine, brain
PXR	LCA, CDCA	Liver, intestine, brain, retina (RPE cells)
VDR	CDCA, CA, LCA	Intestine, brain retina, kidney, retina, bone
GR	UDCA TCA GDCA TUDCA	Liver, brain, retina
CAR	LCA	Liver, brain, kidney, adrenal
**Membrane Receptor**	**Affinity**	**Location**
TGR5	LCA, DCA, CDCA, CA	Liver, intestine, brain, eye (primary retina ganglion cells) spleen, lung, monocytes
S1PR2	TCA, DCA, TDCA, GDCA, TUDCA	Liver, brain, eye, lung, ear
α5β1	TUDCA	Liver brain, retina

## Data Availability

Not applicable.
